# Patients’ Experiences of the Treatment Received During Their Stay in the Stroke Unit at Spanish Healthcare Centers: A Qualitative Approach

**DOI:** 10.3390/medicina61071185

**Published:** 2025-06-29

**Authors:** Sagrario Pérez-de la Cruz

**Affiliations:** Department of Nursing, Physiotherapy and Medicine, University of Almería, 04120 Almería, Spain; spd205@ual.es

**Keywords:** stroke, qualitative research, physiotherapy, treatment, healthcare, relationship

## Abstract

*Background and Objectives*: Stroke is a condition that seriously impairs the personal, family, social and professional lives of those affected. The aim of this study was to explore the perceptions of patients who have experienced a stroke and received care in stroke units across various health centers in Spain, as well as to gain insight into their rehabilitation process. *Materials and Methods*: A qualitative study was carried out with individual interviews involving 30 patients who voluntarily wished to collaborate, recounting their personal experience of the treatment and professionalism with which they were treated in these units. *Results*: Four themes emerged in this study: healthcare, the role of health professionals in the rehabilitation process, the professional–patient relationship, and barriers and facilitators of this relationship. The participants showed the variability in the care protocols for acute stroke patients, as well as the importance of a close relationship with physiotherapists and the demand for professionalism. *Conclusions*: The heterogeneity in the implementation of action protocols by healthcare professionals in stroke units has been demonstrated, leading to inequality in the approach and treatment of these patients. These findings may be useful to identify ways of approaching acute patients in stroke units and to design a comprehensive and coordinated rehabilitation program for these patients.

## 1. Introduction

Stroke is a cerebrovascular disease with significant health and social ramifications due to its high incidence and prevalence. It is regarded as the primary cause of acquired disability in adults in developed countries. Its prevalence increases progressively after the age of 40, affecting more than 10% of the population aged 85 years or older, with an adjusted prevalence of 15.3 cases per-1000 population, being higher in men than in women (18.3 vs. 12.9) [1,2]. In Spain, the National Institute of Statistics reports that approximately 500,000 individuals are living with acquired brain injury (ABI), with 78% of these cases resulting from stroke, which is the leading cause of ABI [3]. Current estimates indicate that between 50,000 and 75,000 people are currently affected by these conditions annually in Spain [3,4].

Initial care in stroke interventions generally commences with the activation of the emergency response system by dialing 061 or 112, or by seeking immediate medical attention at the emergency department. The importance of rehabilitation during the acute phase stems from the existence of a therapeutic window, a period in which intervention can change the course of the disease and cause neuronal reactivation. This improvement may be attributed to the presence of a penumbra zone surrounding the ischemic core. The reperfusion of neurons in this area can lead to a reversal of the initial damage within a relatively short period of up to 24 h and may therefore be reversible [5,6]. Physiotherapy treatment should be initiated after the assessment of premorbid status in patients with the ability to participate in active rehabilitation (Level of evidence A), and within an active and complex stimulating environment [7,8,9]. Patients who initiate physiotherapy treatment during the first week post-stroke demonstrate reduced disability and higher long-term quality of life than those who commence treatment at a later stage [10]. The AVERT and AMBOES studies, which examined the effects of early mobilization interventions, revealed a higher incidence of complications in very early mobilization, advising intervention initiation after the first 48 h [11,12].

In Spain, the Stroke Strategy of the National Health System [13] and, specifically, the regional plans in the autonomous communities establish organizational systems adapted to implement these advances in clinical practice, based on the recommendations of scientific societies [14,15]. These plans propose the implementation of stroke units (SUs) as the most effective and efficient healthcare resource for the treatment of stroke patients, as they benefit the largest number of individuals, reducing mortality, dependence and the need for institutionalization [7,12]. The implementation of the stroke code has been instrumental in increasing the number of patients receiving specialized care and reducing treatment delays, thereby enhancing outcomes and significantly mitigating the impact of this condition [16,17]. However, considerable variations in the implementation of stroke care plans persist among the various autonomous communities. Their implementation in Spain has been gradual and inconsistent [16].

Despite the increase in studies and systematic reviews examining the effectiveness of rehabilitation [18,19,20], there is a paucity of research that captures patients’ views and experiences of their rehabilitation process at this early stage of their recovery. A recent study by Pindus et al. [21] revealed that both patients and their carers feel neglected by public services and face difficulties in reintegrating into their daily activities. More qualitative studies are needed to gain an adequate understanding of the impact of therapies from the perspective of those living with the disease.

In this context, qualitative research can provide valuable insights into the complexities of optimizing therapeutic judgements and inform the design of appropriate interventions. It is recommended that researchers collaborate with people who have had direct experience with certain health conditions to understand the key factors that influence appropriate and tailored therapy programming, in order to bridge the gap between knowledge and clinical practice [22].

The present study was designed to examine the perceptions of patients who received direct care in stroke units of healthcare centers during the acute phase of their condition, focusing on the interpersonal professional treatment of healthcare staff and its impact on their rehabilitation process.

## 2. Materials and Methods

### 2.1. Sample/Participants

The participants in this study were individuals with chronic stroke. All subjects provided a medical report confirming their diagnosis, thereby meeting the study’s eligibility criteria. Inclusion criteria required participants to be between 18 and 75 years of age, to have experienced a stroke with a chronic progression of at least two years, to have received treatment in stroke units in their respective cities, and to be Spanish-speaking. Informed consent was obtained from all eligible participants prior to their definitive inclusion in the study. For those unable to physically sign the consent form, verbal authorization was recorded during the interview. This recorded consent was considered equally valid as the written form. The exclusion criteria included patients with acquired brain damage not attributable to stroke, whose consciousness, cognitive abilities and/or language precluded verbal communication.

A chain sampling approach was adopted for participant selection. It started with a diverse initial sample (contact with health centers) and was combined with a simple random sampling technique, as long as they met the inclusion criteria of the study. The sample formation was complemented by snowball sampling (in order to increase the number of subjects), trying to minimize the likely recall bias. To this end, only centers treating patients who have suffered brain damage and were admitted during their acute phase were contacted. Using this method, a final sample of 30 individuals was obtained.

### 2.2. Study Design

The present study employs a qualitative approach, with an interpretive orientation to the collection and analysis of the data obtained [23]. Data collection was carried out meticulously, using a combination of individual, face-to-face, and/or telephone interviews. In cases where a participant was unable to attend the face-to-face interview, they were offered the opportunity to conduct the interview via telephone [24]. In this manner, in-depth, semi-structured interviews were conducted, thereby fostering enhanced comprehension and a sense of trust among the participants. This approach enriched the quality of their contributions and opinions. The Consolidated Criteria for Reporting Qualitative Research (COREQ) checklist was used for the design of this study [25].

### 2.3. Data Collection

**Figure 1:**
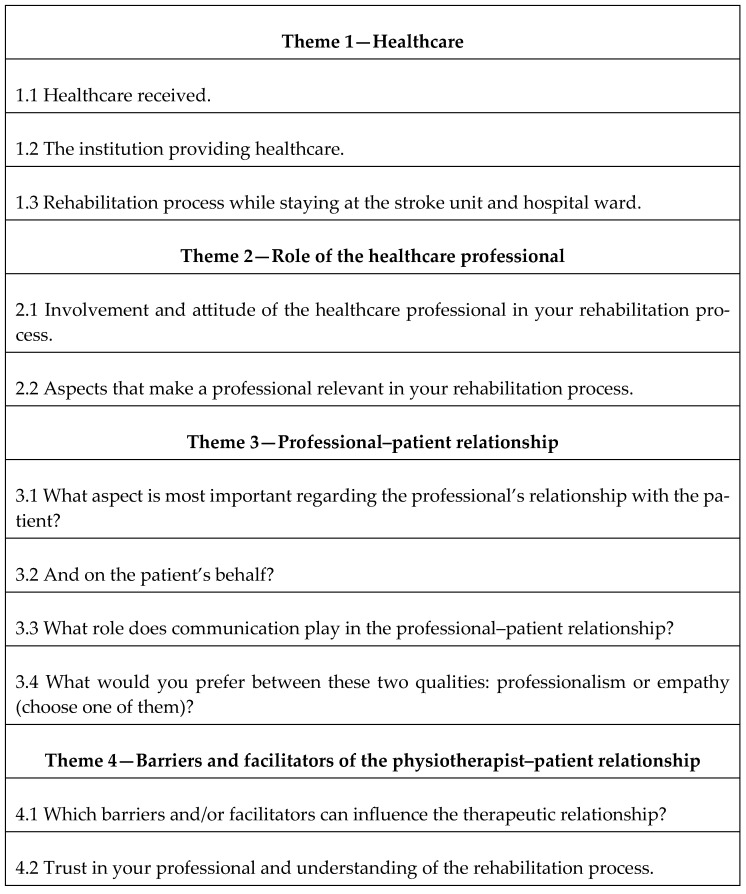
Interview guide.

Data collection was carried out between June and early December of 2024. This phase was conducted in settings considered comfortable for the participants, with locations spanning various geographical regions across the country, including central and southern Spain. Several interviews were conducted by telephone due to the physical distance between the interviewer’s base and the participants’ locations. All interviews were conducted in Spanish and lasted between 15 and 45 min. A semi-structured interview guide, developed based on observations and findings from previous related studies (Figure 1), was used to explore four key areas in depth. The guide was carefully reviewed and refined to ensure the collection of rich and relevant information.

To minimize bias, the interviewer maintained a neutral stance and refrained from interacting beyond what was necessary for the interview process. With participants’ consent, all sessions were recorded to ensure accurate documentation of verbal responses, non-verbal cues, and the overall interaction dynamics [18]. Socio-demographic information was collected at the beginning of each interview.

### 2.4. Analysis of the Results

Audio recordings of the interviews were transcribed verbatim, replacing personal data with ordinal numbering. These recordings and their transcripts were checked again for accuracy. The analysis of the recordings was conducted through the framework approach (also known as qualitative content analysis), allowing descriptive or explanatory conclusions to be drawn, grouped according to specific themes. Before establishing a thematic structure, the reviewer familiarized herself with the interviews by listening to the recordings and reviewing notes taken during the sessions (treatment received, attitude of the professionals, barriers and facilitators of communication, etc.). To create a coding system, a number of interviews were randomly reviewed and code sets applied to the study were defined. The process of constructing, reviewing and defining themes was carried out by the lead author (SPC). During transcription and data entry for analysis, an external professional statistician supervised the entire process. In cases of disagreement between the principal investigator and the statistician, an independent third party was consulted to mediate and resolve the issue. The final themes and sub-themes were discussed and agreed upon with several external experts via video conference, providing guidance and valuable feedback to shape the study’s direction. All interviews were coded using ATLAS-TI 7.1 software, and the codes were organized by themes.

Subsequently, code groupings were defined, followed by the final step of interpreting the data. The lead author met twice with the external statistician to review the characteristics and differences in the data.

### 2.5. Ethical Issues/Statement

This study was approved by the Bioethics Committee of the University of Almeria (Spain) under reference UALBIO2023/045. To participate in this research, the individuals had to provide informed consent, after being duly informed by the investigator about the objectives and characteristics of the study.

## 3. Results

The mean age of the participants was 44 years (range 19–67), and the mean duration of their disease was 4.2 years (range 12 months–9 years). The geographical location of the patients was distributed between central and southern Spain.

The following section presents an analysis of the results obtained from these interviews, capturing the participants’ first-person experiences and perspectives.

### 3.1. Theme 1: Healthcare

Patients with neurological conditions require physiotherapy to recover and/or maintain as much function as possible following neurological damage. However, the nature of the neurological physiotherapy treatments varies among patients, influenced not only by individual needs but also by differences in healthcare services across regions and the patients’ family circumstances. Three sub-themes emerged: Healthcare received, the institution providing health care, and the rehabilitation process during the stay at the stroke unit and on the ward.

#### 3.1.1. Subtheme 1.1: Healthcare Received

The healthcare received by each patient is unique and varies from the experiences of others. While some patients relied exclusively on public services for their treatment, others began with treatment in public services and, upon completing that phase, sought private care (although this was not the focus of the study). In relation to the rehabilitation process during the stay in the stroke unit and on the ward, in general terms, the treatment received was centered on care in the physiotherapy gymnasium. The presence of the physiotherapist in the stroke units is very scarce (if they are present at all, their treatment is occasional and there is little direct care), and this is also the case on the ward when they are admitted.

‘On 9 March I started with a loss of strength in my right arm and was diagnosed with an anxiety attack. I went home and in a couple of days I felt worse and ended up in the emergency room of the XX hospital where they determined that I had suffered a stroke. I was admitted to the stroke unit for 4 days, but when I left everything was complicated by a compartment syndrome, due to a hematoma, which meant that everything was paralyzed, and I went back to the operating theatre. I only had therapy for the first week because it stopped due to the operation. The total stay in the XX was one month.’ (P 3).

‘After a month I was referred to the XX clinic (medium stay brain damage) in mid-April. There I had physiotherapy, occupational therapy, speech therapy and neuropsychology from Monday to Friday (as it was a service provided by the health service of her autonomous community). After two months, I was transferred to an outpatient regime, with two hours a day of therapy, one of physiotherapy and the other shared with the rest of the professionals.’ (P 10).

‘My accident happened in August, and I was admitted to the ICU for a couple of days until it stabilized. During that time nobody came to give me rehabilitation. I only had (occasional) contact with nurses and once a day the doctor came. Then I went up to the ward and was admitted to hospital X for 30 days. I can say that they didn’t pay any attention to what I asked for (a lot of pain in my shoulder and sacrum, and the nurses told me to put up with it). In all that time the physio only came up one day, and all he did was move my arms and legs. I told him that my shoulder was hurting, and asked if he could help me, however he just left.

‘A couple of days before I was discharged (and without knowing what would become of me because I could not fend for myself), they told me that they would talk to a center in X (a city in the same autonomous community, although very far away) to assess me and see if I was a candidate for treatment at that center. What a horrible day I had! They hurt me a lot and I could see that their assessment was ridiculous. Before we left (all the professionals who were there assessed me in an hour and a half), they told me (both my family and myself) that there was no solution and that from now on I should ‘fend for myself’. I’m honestly not lying. What I am telling you can be confirmed by my husband, my sister and my son, who were present.’ (P 22).

#### 3.1.2. Subtheme 1.2 and 1.3: The Institution Providing Healthcare and Rehabilitation Process During Their Stay

For patients belonging to the same autonomous community, the care protocol was similar, although this was not the case for patients from neighboring communities. One of the differences was the length of hospital admission and patient discharge criteria.

In some cases, at the time of admission for the stroke, they were treated in large hospitals with a stroke unit; however, the duration of their stay was variable, and they were then referred to specific medium-stay treatment centers specializing in acquired brain injury.

In contrast, other communities may claim to have stroke units (not in all cities), but both the specific treatment referred to the unit and the subsequent rehabilitation treatment are carried out in the same center, and even by the same professionals who attend rehabilitation both in the unit as well as in the ward and gymnasium.

‘Initially I was referred to the XX (public hospital) for a month. I only had physiotherapy, but very little. Then I was at the XX hospital (public half-stay hospital) for 3 months, with daily intensive treatment, receiving physio and occupational therapy. Then I went to Ceadac (a public non-health center specialized in neurological damage) for 7 months, and I ended up at XX (private center) since October 2019.’ (P 6).

‘It hit me at home at night, and I fell to the floor. I arrived at the hospital by ambulance, they did tests in the emergency room. The treatment was fantastic (the hospital is in the north of Spain). I was not in the stroke unit, and they operated on me. I spent four days in the boxes and got worse because I had another stroke. All together I was there one week and then I was discharged (even though my left side was paralyzed).

Then, I had four months of outpatient physiotherapy treatment, but it sucked. I wanted to do it on my own at home, without professional guidance.

In occupational therapy I did make progress (received after physiotherapy). There, I really did improve. Thanks to a relative, I got to know a private neurological center.’ (P 19).

### 3.2. Theme 2: Role of Health Professionals

Overall, the involvement and attitude of the health professionals in the recovery process of the patient who suffered neurological damage was positive, with particular emphasis on their personal qualities rather than solely their professional expertise. However, many participants noted a lack of specialized training among these professionals in neurological physiotherapy, which can affect the quality of care, despite their work within stroke units in public health centers [2,3,6].

#### 3.2.1. Subtheme 2.1: Involvement and Attitude of the Healthcare Professional

‘Physiotherapists are important, but not essential or necessary’ (P 2); very good treatment’ (P 8). ‘I have been fortunate (public hospital) because the physiotherapist I had was in charge of neurology, because when Christmas came, the physiotherapists who replaced her made me notice the difference and the change. They tried their best, but they were not professional’ (P 9). ‘They had a very good attitude, but they didn’t know how to deal with it.’ (P 27).

#### 3.2.2. Subtheme 2.2: Aspects That Make a Professional Relevant in Your Rehabilitation Process

When the participants were asked about the aspects that they believed to be relevant in the rehabilitation process, the responses were variable. However, the majority concurred in acknowledging the good work of the rehabilitation provided, with the following ideas being particularly salient: ‘Good treatment and I notice that it means I am improving’ (P 13). ‘to perceive that they are “good professionals”, when it comes to touching me, to feel that they know how to do it or that they aren’t fearful. For them to show that they are confident in their handling and explanations.’ (P 22). ‘Good treatment, kind, that does me little harm.’ (P 3). ‘A good professional, who shows empathy with patients, to be treated gently and energetically, with kindness.’ (P 29).

### 3.3. Theme 3: Professional-Patient Relationship

#### 3.3.1. Subtheme 3.1 and 3.2: Professional Relationship with the Patient

For the patients, the qualities that a good professional should possess are diverse, although in summary they requested a balance between the personal and professional spheres: ‘to have the upper hand, because our behavior makes it necessary for the physio to know how to lead, direct and work” (P 1); ‘empathy’ (P 4); ‘to administer good personal treatment’ (P 5); ’firstly: to be close, to explain things in a simple manner. Secondly: to have knowledge of neurological physiotherapy, because I have had previous experiences that were not so pleasant.’ (P 11). These are examples of qualities that patients request from their professionals.

Conversely, in the alternative scenario (regarding their beliefs of what a professional may define as a ‘good patient’), the following qualities were suggested: ‘they have to trust the professional’ (P 30); ‘be very willing to work’ (P 18); ‘be obedient and be able to reach a common agreement when it comes to planning the treatment’ (P 20); ‘not be very serious, let yourself go and do what you are told.’ (P 23). A common defining trait was that patients should work in harmony with the professional.

#### 3.3.2. Subtheme 3.3: Communication

Regarding the communication that should exist between patients and professionals, there was unanimous agreement that this is a ‘very important element’ (P 3, P 11, P 15, P 27) and even ‘essential’ (P 4, P 6, P 14, P 20).

#### 3.3.3. Subtheme 3.4: Professionalism or Empathy

In the context of this theme, one of the issues that was addressed with the participants concerned the idealization of their profession. The prevailing paradigm concerning the hallmarks of a competent professional is encapsulated by the juxtaposition of two qualities: professionalism and empathy. When presented with the conundrum of ascribing greater significance to one over the other, most of the participants exhibited a slight predilection for professionalism, while the remainder demonstrated a preference for empathy as the predominant characteristic. However, all participants expressed a strong desire to encounter a professional who could embody both qualities.

### 3.4. Theme 4: Barriers and Facilitators of the Physiotherapist–Patient Relationship

A plethora of barriers were identified by the participants as hindering their relationship with their respective physiotherapists, including ‘pain felt during treatment’ (P 15); ‘lack of personal interest of the physiotherapist in the patient, and vice versa’ (P 27); or ‘being surly’ (P 29). Conversely, factors conducive to more harmonious relationships were also identified, such as ‘closeness’ (P 29); ‘pleasant atmosphere’ (P 30); and ‘both being hard workers’ (P 9).

Finally, it was important to explore whether patients consistently felt confident in the professionals treating them during their stay in the stroke units, and whether any doubts they had about the proposed treatments were adequately addressed. While most participants reported feeling confident in their healthcare providers, this confidence often did not extend to a clear understanding of the therapeutic approach being applied. This lack of clarity was attributed to several factors: some patients did not ask for further information (P 1, P 5, P 17); others perceived a lack of expertise in neurological physiotherapy among professionals (P 8, P 11, P 19, P 26); and in some cases, explanations were directed only toward student trainees rather than the patients themselves (P 18).

## 4. Discussion

The main objective of this study was to analyze the perception of patients who had experienced a cerebrovascular accident (stroke) and who had received treatment in national stroke units for their specialized care, recognizing that physiotherapy plays a fundamental role in the rehabilitation of patients with acquired brain injury (ABI) [19]. According to one of the studies reviewed, these patients receive physiotherapy care in their referral hospitals. However, a significant number of them choose to seek more specialized treatment in private clinics, motivated by long waiting lists in public services [20]. Recent studies have shown a negative correlation between increased time from discharge from the intensive care unit to admission to rehabilitation programs, resulting in clinical outcomes that cannot be explained solely by the initial severity of the injury [21,22]. Furthermore, it has been documented that patients who start rehabilitation in the intensive care unit have better outcomes compared to those who do not [22].

- Healthcare:

It is concerning that many stroke survivors feel abandoned and marginalized by health services. Lack of continuity in care and active follow-up are critical shortcomings that can significantly affect the recovery of these patients. The absence of adequate and specific information regarding their condition and the resources available further contributes to the sense of disorientation experienced by these individuals, leaving them without the necessary tools to meaningfully engage in their recovery process.

Another key issue that affects patients who have suffered a stroke is related to the training of health professionals and the availability of scientific evidence supporting the physiotherapist’s role within stroke units. Strengthening this aspect is essential to improving care. Providing consistent professional support, particularly during the first year post-stroke, could empower patients to manage their chronic condition more effectively [23,24,25,26]. The provision of clear and accessible information, specific training plans, and the establishment of regular follow-ups would not only foster a greater sense of support for patients but also facilitate the development of long-term relationships of trust with health professionals. This recommendation could be implemented through mandatory hospital training sessions for staff and the implementation of unified and scientifically evidenced protocols on the comprehensive and appropriate management of stroke patients in all phases of their evolution. It is an endeavor that merits exploration with a view to enhancing the quality of life of those who have endured this arduous experience. Therefore, it is essential that the information provided by the professional is clear, accessible and reliable. This would not only enhance health literacy regarding stroke but also promote improved self-management of the condition [19,21]. In turn, this could lead to the greater and more effective involvement of all stakeholders involved in the rehabilitation process.

- Care provided by health professionals

In addition, participants described the need to be listened to and to receive individualized care and rehabilitation [26,27,28,29]. Listening to and considering the patient as a person capable of participating in decision making and planning their own care and rehabilitation fits into the person-centered approach [30,31]. Some of the statements made by participants in this study are consistent with the concept of person-centeredness, which is supported by national stroke guidelines [32]. A relevant aspect, according to patients’ perceptions, is the importance of professionalism and empathy on behalf of the health professionals who care for them [19]. Physiotherapists focus on providing rehabilitative treatments to patients with acquired brain injury (ABI) [33,34]; however, they often underestimate the impact they have as professionals who interact with these individuals on a prolonged basis [35,36]. This can lead to disregard for patients’ emotions and a lack of involvement in their complex acceptance process. Furthermore, there is insufficient evidence to document this dynamic in relation to patients’ future aspirations. This study is essential for professionals in the field, as it offers a perspective on how patients with ABI perceive their disease and the recovery process from the field of physiotherapy. This information can facilitate the implementation of a more comprehensive and humanized therapeutic approach.

### Limitations

This study provides valuable new insights into the dilemmas faced by stroke survivors and their experience in stroke units and physiotherapy rooms in public hospitals. However, it is important to discuss several limitations that could affect the generalizability of our findings. One of the first and important limitations could be that it was conducted by a single author, which could introduce interpretative bias. However, to mitigate this risk, two external professionals were consulted throughout the analysis process to enhance the validity and objectivity of the findings. Second, data were collected only from patients who received treatment in central and southern Spain, which limits the geographical representativeness of the study. It would be advisable to broaden the geographical scope of the study to include and compare the different healthcare systems implemented across the country. Third, all participants received care in urban areas, where healthcare resources tend to be of higher quality and more readily accessible compared to rural settings. This suggests that our results may not adequately reflect the specific needs and difficulties of stroke survivors in regions where the implementation of hospital-based stroke units has been less developed. In addition, it would have been interesting to explore other aspects related to the entire rehabilitation process, including economic, social and family factors. These factors influence, both directly and indirectly, patients’ perceptions of their recovery after a stroke. Incorporating these dimensions could provide a more holistic and comprehensive understanding of the rehabilitation process, helping to identify additional areas for improving patient care. It is also necessary to recognize the limitations in generalizing the results obtained, proposing the extension of the geographical area of intervention.

## 5. Conclusions

This research highlights the multiple difficulties faced by adult stroke survivors, especially regarding access to acute care services in public facilities. Lack of adequate information about available services exacerbates these difficulties, and factors dependent on the professionals themselves often hinder timely help-seeking. The findings underline the critical need for a coordinated approach to improve access to rehabilitation and care in the short to medium term. Increasing accessibility to medical resources, improving support for daily activities, and more effective dissemination of health information and neurological-specific training by physiotherapists are essential. To address these challenges, we propose an integrated care pathway that ensures continuous support.

Governments, healthcare providers, and communities must work together to create environments that support care for stroke survivors, effectively addressing the needs of both patients and the professionals who care for them. This information can be essential to develop more comprehensive healthcare solutions tailored to the specific needs of each region. By addressing these aspects, the quality of life of stroke survivors and healthcare professionals can be significantly improved, ensuring a more equitable, professional, and accessible care system for all.

## Data Availability

The original contributions presented in this study are included in the article. Further inquiries can be directed to the corresponding author.

## Abbreviations

The following abbreviations are used in this manuscript:

COREQ	Consolidated Criteria for Reporting Qualitative Research
ABI	Acquired Brain Injury
SU	Stroke Units
ICUs	Intensive Care Units
